# Commentary on Special Issue: “Towards a Biocultural Synthesis of the Peopling of the Americas”

**DOI:** 10.1002/ajpa.70149

**Published:** 2025-11-03

**Authors:** Maria A. Nieves‐Colón

**Affiliations:** ^1^ Department of Anthropology University of Minnesota Twin Cities Minneapolis Minnesota USA

The peopling of the Americas has been a major topic of study in Anthropology since the early days of our field. However, the emergence of new technologies and theoretical frameworks, plus the growth of decolonial and engaged approaches has prompted a revision of previous understandings (Menéndez et al. 2022; Raff 2022; Willerslev and Meltzer 2021). The contributions in this special issue “Towards a biocultural synthesis of the peopling of the Americas” illustrate this pivotal redirection, providing readers with a comprehensive view of how biological anthropologists today are rethinking what we thought we knew about the first communities to inhabit the double continent.

Many studies in this special issue re‐engage and revisit “classic” lines of evidence (e.g., linguistics, dental and cranial morphology, kinship studies, mitochondrial DNA), but this move is not nostalgic or wistful. Instead, it seeks to test, refine, and modernize these frameworks with new data, methods, and an interdisciplinary perspective. For example, anthropologists have long theorized that kinship and postmarital residence patterns are essential aspects of human social life (Cveček 2024). Figueiro (2025) connects kinship theory with paleogenomics by using computational simulations to test how ancient kin systems may have shaped the mtDNA diversity patterns seen in burial contexts. This study reminds us that kinship is not solely “ethnographic context” for population genetics studies but instead a key variable for comprehensive anthropological inference.

Similarly, Nichols (2025) and Scott et al. (2025) also revisit established methodologies and long‐standing debates through novel and interdisciplinary frameworks. Nichols (2025) uses graphic cluster analyses to investigate the diversity and typological features of Indigenous American languages in relation to genetic, paleoclimatic and archaeological data. While Scott et al. (2025) apply statistical methods borrowed from forensic science to a large comparative dataset of dental morphological traits. Their biodistance analysis reconstructs demographic processes and characterizes ancestral relationships between Indigenous Americans and East Asian populations. Both studies build upon longstanding areas of anthropological inquiry while displaying deep respect for the previous generation of scholars who laid the theoretical and methodological foundation for today's work (Greenberg et al. 1986; Turner 1983). The contrasting patterns that each study identifies—Nichols (2025) finds support for multiple initial entries into the Americas, while Scott et al. (2025) propose a single migration model—illustrate both the promise and challenge of integrating multiple lines of evidence to address complex questions. Indeed, as prior scholarship is revised with today's more interdisciplinary methods, the scholars in this issue find that multiple and sometimes contrasting explanations are required to make sense of the complex patterns of variation that they observe.

Importantly, the studies in this collection reject simple models and straightforward explanations and instead challenge us to think more deeply and creatively about the heterogeneity and diversity of early American populations. Using multi‐isotope analysis, Chinique de Armas et al. (2025) demonstrate diverse dietary and mobility patterns among early societies in Cuba. Their findings reject prior cultural‐historical models, which characterized early peoples as static and uniform communities. Instead, the authors propose a more complex vision of early Caribbean societies as regionally varied, dynamic and pluralistic. Similarly, Arencibia et al. (2025) conduct a detailed analysis of two B2 mitogenomes found among individuals from a single burial context in ancient Patagonia. Their findings suggest that the Patagonian B2 clade was more diverse in the past than it is today, indicating that the peopling of the Southern Cone was a more complex and lively process than we previously envisioned.

Several contributions also highlight the continued relevance of non‐genetic methods even in our current “paleogenomics revolution” moment (Callaway 2023). Similar to other studies in this issue (see Figueiro 2025 and Chinique de Armas et al. 2025), the osteological analyses conducted by Smith‐Guzmán et al. (2025) prompt us to rethink burial spaces and their use and illustrate the value of thinking outside of long held theoretical paradigms. Using dental biodistance analyses they find that collective burials in Panama represent community burial grounds that hold individuals from nearby villages and not extended family members as previously thought. This study also signals how biodistance analyses can fill important gaps left by poor ancient DNA preservation, limited genetic sampling of modern populations, or situations where destructive analysis is not a feasible or ethically sustainable option.

Nearly all the studies emphasized the need for a multidisciplinary approach and integration of multiple lines of evidence. Castro E Silva and Hünemeier (2025) demonstrate the power of this approach in a comprehensive piece that reviews archaeological, linguistic, and genetic evidence for the demic expansion of Tupi‐speaking peoples across the Amazon and broader South America. Similarly, by combining multiple threads of evidence, Menéndez and Urban (2025) track parallel evolutionary processes in linguistic and morphological structures among understudied populations from the Southern Cone. These studies reflect a core theme of this special issue: that no single dataset is sufficient to capture the biocultural complexity of the early populations of the Americas.

Finally, this issue also provides a snapshot of the multi‐institutional and international nature of modern biological anthropology research. The articles have a diverse author pool, with a mix of senior and emerging scholars, reflecting a diversity of training backgrounds, subfield specialties and career stages. Notably the works gathered here also demonstrate substantial Latin American leadership with many first or corresponding authors hailing from countries that until recently were poorly represented in these positions. The articles gathered here reflect a growing trend towards greater inclusivity, capacity building, and regional leadership as local scholars team up with international colleagues in collaborative partnerships. This is a welcome change for a field whose scholarship was until recently mostly dominated by Global North perspectives.

Taken together, the special issue presents a picture of the peopling of the Americas as a mosaic of processes, some local, some regional, and others occurring at continental scales. It also points towards areas of interest for future research. For example, how will the integration of emerging methodological approaches such as paleoproteomics (Warinner et al. 2022) and machine learning or artificial intelligence (Lye et al. 2024; Pless et al. 2023) change our understanding of the early settlement of the Americas? What areas of the Americas are still underexplored and in need of attention? And how will biological anthropologists deal with the ethical issues that our work raises for the diverse Indigenous and descendant communities that live across the American continents? With emerging scholarship like the one in this collection, I for one am excited to find out.

## Author Contributions

**Maria A. Nieves‐Colón:** conceptualization, writing – original draft, writing – review and editing.

## Data Availability

Data sharing not applicable to this article as no datasets were generated or analysed during the current study.

## References

[ajpa70149-bib-0001] Arencibia, V. , M. Muñoz , C. M. Crespo , et al. 2025. “Novel B2 Mitogenomes From Continental Southern Patagonia's Late Holocene: New Insights Into the Peopling of the Southern Cone.” American Journal of Biological Anthropology 186, no. 1: e24822. 10.1002/ajpa.24822. 37548135

[ajpa70149-bib-0002] Callaway, E. 2023. “‘Truly Gobsmacked’: Ancient‐Human Genome Count Surpasses 10,000.” Nature 617: 20. 10.1038/d41586-023-01403-4. 37095409

[ajpa70149-bib-0003] Castro E Silva, M. A. , and T. Hünemeier . 2025. “A Multidisciplinary Overview on the Tupi‐Speaking People Expansion.” American Journal of Biological Anthropology 186, no. 1: e24876. 10.1002/ajpa.24876. 37990807 PMC11774006

[ajpa70149-bib-0004] Cveček, S. 2024. “Why Kinship Still Needs Anthropologists in the 21st Century.” Anthropology Today 40, no. 1: 3–6. 10.1111/1467-8322.12861.

[ajpa70149-bib-0005] Chinique de Armas, Y. , W. M. Buhay , U. M. González Herrera , et al. 2025. “Diversity of Lifeways in Early Antillean Societies: A Multi‐Isotope Approach.” American Journal of Biological Anthropology 186, no. 4: e70039. 10.1002/ajpa.70039. 40241353 PMC12003967

[ajpa70149-bib-0006] Figueiro, G. 2025. “Simulating the Effects of Kinship and Postmarital Residence Patterns on Mitochondrial DNA Diversity in Mortuary Contexts.” American Journal of Biological Anthropology 186, no. 1: e24910. 10.1002/ajpa.24910. 38317510

[ajpa70149-bib-0007] Greenberg, J. H. , C. G. Turner , S. L. Zegura , et al. 1986. “The Settlement of the Americas: A Comparison of the Linguistic, Dental, and Genetic Evidence [and Comments and Reply].” Current Anthropology 27, no. 5: 477–497. 10.1086/203472.

[ajpa70149-bib-0008] Lye, R. , H. Min , J. Dowling , et al. 2024. “Deep Learning Versus Human Assessors: Forensic Sex Estimation From Three‐Dimensional Computed Tomography Scans.” Scientific Reports 14, no. 1: 30136. 10.1038/s41598-024-81718-y. 39627517 PMC11615306

[ajpa70149-bib-0009] Menéndez, L. P. , K. S. Paul , C. de la Fuente , et al. 2022. “Towards an Interdisciplinary Perspective for the Study of Human Expansions and Biocultural Diversity in the Americas.” Evolutionary Anthropology: Issues, News, and Reviews 31, no. 2: 62–68. 10.1002/evan.21937. 35043498

[ajpa70149-bib-0010] Menéndez, L. P. , and M. Urban . 2025. “Tracing Human Diversity in South America's Southern Cone: Linguistic, Morphometric, and Genetic Perspectives.” American Journal of Biological Anthropology 187, no. 3: e70077. 10.1002/ajpa.70077. 40579880 PMC12205295

[ajpa70149-bib-0011] Nichols, J. 2025. “Founder Effects Identify Languages of the Earliest Americans.” American Journal of Biological Anthropology 186, no. 1: e24923. 10.1002/ajpa.24923. 38554027 PMC11775432

[ajpa70149-bib-0012] Pless, E. , A. M. Eckburg , and B. M. Henn . 2023. “Predicting Environmental and Ecological Drivers of Human Population Structure.” Molecular Biology and Evolution 40, no. 5: msad094. 10.1093/molbev/msad094. 37146165 PMC10172848

[ajpa70149-bib-0013] Raff, J. A. 2022. Origin: A Genetic History of the Americas. Hachette Book Group.

[ajpa70149-bib-0014] Scott, G. R. , D. Navega , T. Vlemincq‐Mendieta , et al. 2025. “Peopling of the Americas: A New Approach to Assessing Dental Morphological Variation in Asian and Native American Populations.” American Journal of Biological Anthropology 186, no. 1: e24878. 10.1002/ajpa.24878. 38018312

[ajpa70149-bib-0015] Smith‐Guzmán, N. E. , J. Smid Núñez , J. D. Cybulski , and L. A. Sánchez Herrera . 2025. “Biodistance Analysis via Dental Phenotypic Diversity in Early Collective Burials at Cerro Juan Díaz, Panamá (30–650 CE ) [Análisis de Biodistancia Mediante la Diversidad Fenotípica Dental en los Entierros Colectivos Tempranos del Cerro Juan Díaz, Panamá (30–650 EC )].” American Journal of Biological Anthropology 186, no. 1: e25050. 10.1002/ajpa.25050. 39704464 PMC11660533

[ajpa70149-bib-0016] Turner, C. G. I. 1983. “Dental Evidence for the Peopling of the Americas.” In Early Man in the New World, edited by R. Shutler , 147–157. Sage Publications (University of Alaska Library, Fairbanks).

[ajpa70149-bib-0017] Warinner, C. , K. Korzow Richter , and M. J. Collins . 2022. “Paleoproteomics.” Chemical Reviews 122: 1c00703. 10.1021/acs.chemrev.1c00703. 35839101 PMC9412968

[ajpa70149-bib-0018] Willerslev, E. , and D. J. Meltzer . 2021. “Peopling of the Americas as Inferred From Ancient Genomics.” Nature 594, no. 7863: 356–364. 10.1038/s41586-021-03499-y. 34135521

